# Editorial: Zoonotic bacteria: genomic evolution, antimicrobial resistance, pathogenicity, and prevention strategies

**DOI:** 10.3389/fvets.2024.1390732

**Published:** 2024-03-08

**Authors:** Abdelaziz Ed-Dra, Filippo Giarratana, Aaron P. White, Min Yue

**Affiliations:** ^1^Laboratory of Engineering and Applied Technologies, Higher School of Technology, Sultan Moulay Slimane University, Beni Mellal, Morocco; ^2^Department of Veterinary Science, University of Messina, Polo Universitario dell'Annunziata, Messina, Italy; ^3^Riconnexia srls, Spin-off of the University of Messina, Polo Universitario dell'Annunziata, Messina, Italy; ^4^Vaccine and Infectious Disease Organization (VIDO), University of Saskatchewan, Saskatoon, SK, Canada; ^5^Department of Biochemistry, Microbiology and Immunology, University of Saskatchewan, Saskatoon, SK, Canada; ^6^Department of Veterinary Medicine, Zhejiang University College of Animal Sciences, Hangzhou, China; ^7^State Key Laboratory for Diagnosis and Treatment of Infectious Diseases, National Clinical Research Center for Infectious Diseases, National Medical Center for Infectious Diseases, The First Affiliated Hospital, College of Medicine, Zhejiang University, Hangzhou, China; ^8^School of Life Science, Hangzhou Institute for Advanced Study, University of Chinese Academy of Sciences, Hangzhou, China

**Keywords:** zoonoses, genomic epidemiology, virulence, pathogenicity, whole genome sequencing, genomic evolution, vaccine development

Zoonotic bacteria pose a significant concern due to their ability to naturally transmit infections from animals to humans. Despite global efforts to improve lifestyle and healthcare accessibility, these bacteria persistently adapt to new challenges, posing an ongoing threat to established health programs and human wellbeing (1). This situation is exacerbated by the emergence of multi-drug resistant strains, which diminish or evade various antimicrobial treatments, highlighting the urgent need for developing new effective and sustainable alternatives (2, 3). Currently, the use of Whole Genome Sequencing (WGS) has revolutionized the surveillance and epidemiologic analysis of zoonotic bacteria, offering unprecedented insights into their genetic makeup and transmission dynamics. WGS allows for the comprehensive examination of an organism's entire DNA, enabling researchers to identify specific genetic markers, trace the outbreak origins, and understand the evolutionary patterns of zoonotic bacteria (4–6).

Animal farms are considered the primary reservoir for developing and spreading harmful zoonotic bacteria. In fact, bacteria that grow in animal farms can cause disease in animals, or be transmitted to humans via direct contact, inhalation of aerosol, or consumption of contaminated animal-derived food products. In this regard, Anjos et al. have used WGS to characterize *Mycobacterium tuberculosis* variant *bovis* (a leading cause of bovine tuberculosis) isolated from cattle circulating in the state of Mato Grosso in Brazil. The WGS analysis showed that the sequenced strains belonged to lineage BOV AFRI and the spoligotype BOV 1; BOV 2, presenting high genetic diversity compared to other Brazilian *M. tuberculosis* var. *bovis* strains, suggesting different transmission routes in the production chain. Additionally, the authors showed that the Brazilian *M. tuberculosis* var. *bovis* harbor genes encoding resistance to different drugs, including pyrazinamide, isoniazid, rifampicin, streptomycin, ethambutol, ethionamide, florquinolones, kanamycin/amikacin, capreomycin, paraminosalicylic acid, cycloserine, bedaquiline, linezolid, and delamanid, with different mutations in the drug resistance encoding genes.

On the other hand, El-Adawy et al. have used WGS to analyze the genetic diversity, resistome, plasmidome, and virulome profiles of 66 *Campylobacter jejuni* isolated between 2010 and 2011 from German turkey flocks. The studied isolates showed high genetic diversity, which were grouped into 28 different sequence types and 11 clonal complexes, with an average pairwise cgSNP distance of 14,585. Additionally, the prediction of virulence genes showed the detection of 30 genes related to motility, chemotaxis, adhesion, and invasion, with the abundance of *flaA* (83.3%) and *flaB* (78.8%) genes encoding for flagellin protein A and B, respectively. The prediction of antimicrobial resistance genes showed the detection of genes encoding resistance toward ampicillin (*bla*_OXA_), tetracycline [*tet*(O)], neomycin [*aph(3*′*)-IIIa*], streptomycin (*aadE*), and streptothricin (*sat4*), in addition to a single mutation T86I in the housekeeping gene *gyrA* conferring resistance to quinolones. The virulence and resistance genes are often carried out by mobile genetic elements, particularly, plasmids in this regard, the authors evidenced the detection of 28 plasmid-borne contigs among the 66 *C. jejuni* isolates. On the other hand, authors showed high resistance to metronidazole (49/66; 74.2%), followed by ciprofloxacin (47/66; 712%), and nalidixic acid (44/66; 66.7%), while all isolates were susceptible to gentamicin, erythromycin, and chloramphenicol.

The main route of transmitting zoonotic bacteria is through the consumption of contaminated animal-derived foods, especially meat products and eggs. Bacteria that belong to the normal flora of many animals can contaminate carcasses during the slaughtering process (7). Rodarte et al. conducted a scoping review on zoonotic parasites and pathogens in Eastern African abattoirs, identifying 42 species affecting workers and slaughtered livestock, notably *Mycobacterium bovis*. In fact, abattoirs are considered a crucial “One Health” interface with frequent interactions between humans, animals, and the environment, requiring improved infrastructure and biosecurity measures. Hence, efforts should address varying hygienic practices across abattoir types, emphasizing personal protective equipment (PPE), training programs, safe animal handling, and vaccination to mitigate zoonotic risks. However, early detection via robust surveillance systems, including WGS-based methods, is imperative to curb pathogen transmission effectively. This underscores the importance of holistic, collaborative approaches, aligning with the One Health approach, to mitigate the spread of zoonotic disease in abattoir settings.

In the absence of effective treatment for zoonotic diseases, early detection is essential to prevent the spread of zoonotic pathogens. Charron et al. have used an integrative genomic and transcriptomic approach to assess the effect of variation in gene content and their expression among *Burkholderia mallei* strains, to evaluate serodiagnostic biomarkers glanders. For this, the authors have analyzed and compared the genomes of *B. mallei* retrieved from both NCBI's RefSeq database and in-house samples with the reference strain *B. mallei* ATCC 23344. The pan-genome analysis identified gene content differences ranging from 31 to 715 (an average of 334 gene presence-absence), with notable losses of genes encoding serodiagnostic antigens due to structural variations. Additionally, the transcriptomic analysis identified 388 differentially expressed genes, including those related to pathogenesis and virulence, influenced by genomic variations. These variations significantly impact host innate and adaptive immunity, particularly antibody production. The study underscores the importance of early detection in zoonotic disease management and highlights the potential for improved glanders serodiagnosis and molecular typing using genomic approaches.

Vaccines have been used for a long time in the veterinary field to prevent disease spread, enhance welfare, reduce treatment costs, and curb zoonotic disease transmission. *Pasteurella multocida* is a zoonotic pathogen that causes pneumonia in a wide range of animals, particularly in pigs, leading to significant economic losses worldwide. Vaccines have been used for over 50 years to prevent and control swine pasteurellosis in China, however, recent studies showed that traditional vaccines had no protective effect against the epidemic serogroup A and D strains. In this regard, Guan et al. assessed the immunogenicity and efficacy of serogroup A and D bacterins against *P. multocida*. For this, 26 inactivated *P. multocida* vaccines were used to target the prevalent serogroups A and D. Immunized mice showed limited homologous protection for genotype A:L6 strains, while some provided heterologous protection against genotype A:L3. Conversely, genotype D:L6 strains offered both homologous and heterologous protection. These findings provided insights into the effectiveness of bacterins as vaccinations against *P. multocida* and provided some baseline references for the development of efficacious bivalent vaccines.

In summary, this Research Topic advances our knowledge of monitoring, diagnosing, controlling, and preventing the emergence of zoonotic bacteria. WGS has proven efficacy as an effective method to enhance the surveillance and diagnosis/detection of virulent and antimicrobial-resistant genotypes, in addition, the development of new vaccines can be considered a key Research Topic for controlling and preventing the development and spread of zoonotic bacteria, especially in the era and antimicrobial resistance.

## Author contributions

AE-D: Writing – original draft, Writing – review & editing. FG: Writing – review & editing. AW: Writing – review & editing. MY: Writing – review & editing.

## References

[1] Li Y Ed-Dra A Tang B Kang X Müller A Kehrenberg C. Higher tolerance of predominant *Salmonella serovars* circulating in the antibiotic-free feed farms to environmental stresses. J Hazard Mater. (2022) 468:129476. 10.1016/J.JHAZMAT.2022.129476 35809365

[2] Ed-Dra A Filali FR Khayi S Oulghazi S Bouchrif B El Allaoui A. Antimicrobial resistance, virulence genes, and genetic diversity of *Salmonella enterica* isolated from sausages. Eur J Microbiol Immunol. (2019) 9:56–61. 10.1556/1886.2018.00035 31223497 PMC6563686

[3] Jia C Wang Z Huang C Teng L Zhou H An H. Mobilome-driven partitions of the resistome in Salmonella. mSystems. (2023) 8:23. 10.1128/msystems.00883-23 37855620 PMC10734508

[4] Zhou X Kang X Chen J Song Y Jia C Teng L. Genome degradation promotes Salmonella pathoadaptation by remodeling fimbriae-mediated proinflammatory response. Natl Sci Rev. (2023) 10:228. 10.1093/nsr/nwad228 37965675 PMC10642762

[5] Feng Y Pan H Zheng B Li F Teng L Jiang Z. An integrated nationwide genomics study reveals transmission modes of typhoid fever in China. mBio. (2023) 14:23. 10.1128/mbio.01333-23 37800953 PMC10653838

[6] Li Y Teng L Xu X Li X Peng X Zhou X. A nontyphoidal Salmonella serovar domestication accompanying enhanced niche adaptation. EMBO Mol Med. (2022) 14:e16366. 10.15252/emmm.202216366 36172999 PMC9641423

[7] Wu B Ed-Dra A Pan H Dong C Jia C Yue M. Genomic investigation of Salmonella isolates recovered from pigs slaughtering process in Hangzhou, China. Front Microbiol. (2021) 12:704636. 10.3389/FMICB.2021.704636 34305874 PMC8298193

